# Supplementary material for: Artisanal fish fences pose broad and unexpected threats to the tropical coastal seascape
Source: Nat Commun. 2019 May 21;10:2100. doi: 10.1038/s41467-019-10051-0 (PMC6529422; doi:10.1038/s41467-019-10051-0)
Supplement: Supplementary file 1 — Supplementary Information [file 41467_2019_10051_MOESM1_ESM.pdf]

Supplementary Information

Artisanal fish fences pose broad and unexpected threats to the tropical coastal seascape

Exton et al.

Supplementary Table 1: Annual time point data for fish fence effort and catch monitoring, showing means ±1SE where applicable.

	EFFORT		CATCH MONITORING				
Year	Total fences in use	Fence length (m; central spine)	No. fences surveyed	No. catches surveyed	CPUE (kg fence^{-1} day^{-1})	Individuals caught (kg fence^{-1} day^{-1})	% juveniles per catch (kg fence^{-1} day^{-1})
2002	37						
2003			5	37	17.45 ±1.64	309.54 ±42.05	
2004							
2005	100	105.7 ±8.9	3	12	18.08 ±6.36	713.50 ±256.67	9.72 ±3.33
2006							
2007	119	168.8 ±5.4	7	75	5.44 ±0.86	172.76 ±34.32	24.06 ±2.28
2008							
2009	210	127.5 ±4.0	17	174	5.81 ±0.33	81.51 ±7.80	28.00 ±1.35
2010							
2011		132.7 ±7.5	18	160	5.26 ±0.33	47.39 ±4.15	46.66 ±1.64
2012	112		7	102	3.65 ±0.22	36.80 ±3.10	36.53 ±2.26
2013		153.0 ±18.9	10	200	6.34 ±0.45	48.43 ±3.42	34.30 ±2.43
2014			8	80	6.22 ±0.60	64.00 ±4.99	44.04 ±1.91
2015	160	174.3 ±3.5	10	92	3.76 ±0.39	52.30 ±5.45	35.23 ±0.34
2016		178.8 ±4.9	11	100	6.29 ±0.76	44.72 ±3.55	40.27 ±1.92

Supplementary Table 2: Changes in the length of ten of the most abundant species from monitored fish fence catches between the start of the study (2003-2004) and the end of the study (2015-2016). Data shown are median length values (cm), with interquartile range and total number of fish measured (*n*) in brackets. Also shown are statistical results for Moods comparison of median tests.

Species	Family	2003-04 length (cm)	2015-16 length (cm)	Moods Test results
Cheilio inermis	Labridae	24.0 (20.0-29.0; *102*)	22.5 (20.6-24.0; *255*)	$X^2 = 0.384$, $p = 0.536$
Gerres oyena	Gerreidae	16.0 (15.0-18.0; *291*)	16.1 (14.1-17.5; *61*)	$X^2 = 0.470$, $p = 0.493$
Lethrinus harak	Lethrinidae	19.0 (18.0-20.0; *303*)	18.1 (13.9-21.8; *174*)	$X^2 = 2.504$, $p = 0.114$
Lethrinus ornatus	Lethrinidae	18.5 (17.0-19.5; *79*)	17.5 (12.2-19.0; *66*)	$X^2 = 0.942$, $p = 0.332$
Lethrinus rubrioperculatus	Lethrinidae	14.5 (12.0-16.0; *409*)	15.6 (14.0-17.4; *222*)	$X^2 = 20.569$, $p = 5.753e^{-6}$
Lethrinus variegatus	Lethrinidae	16.0 (15.0-17.0; *160*)	14.3 (12.5-15.7; *189*)	$X^2 = 32.982$, $p = 9.301e^{-9}$
Parupeneus barberinus	Mullidae	17.0 (16.0-19.0; *117*)	15.6 (13.7-17.0; *76*)	$X^2 = 22.595$, $p = 2.0e^{-6}$
Scolopsis trilineata	Nemipteridae	16.0 (14.0-16.5; *159*)	14.4 (13.2-17.0; *15*)	$X^2 = 0.292$, $p = 0.589$
Siganus canaliculatus	Siganidae	16.0 (15.0-17.0; *858*)	18.1 (15.6-20.6; *570*)	$X^2 = 231.766$, $p < 2.2e^{-16}$
Upeneus tragula	Mullidae	17.0 (16.0-18.0; *19*)	15.1 (13.8-16.8; *46*)	$X^2 = 1.232$, $p = 0.267$

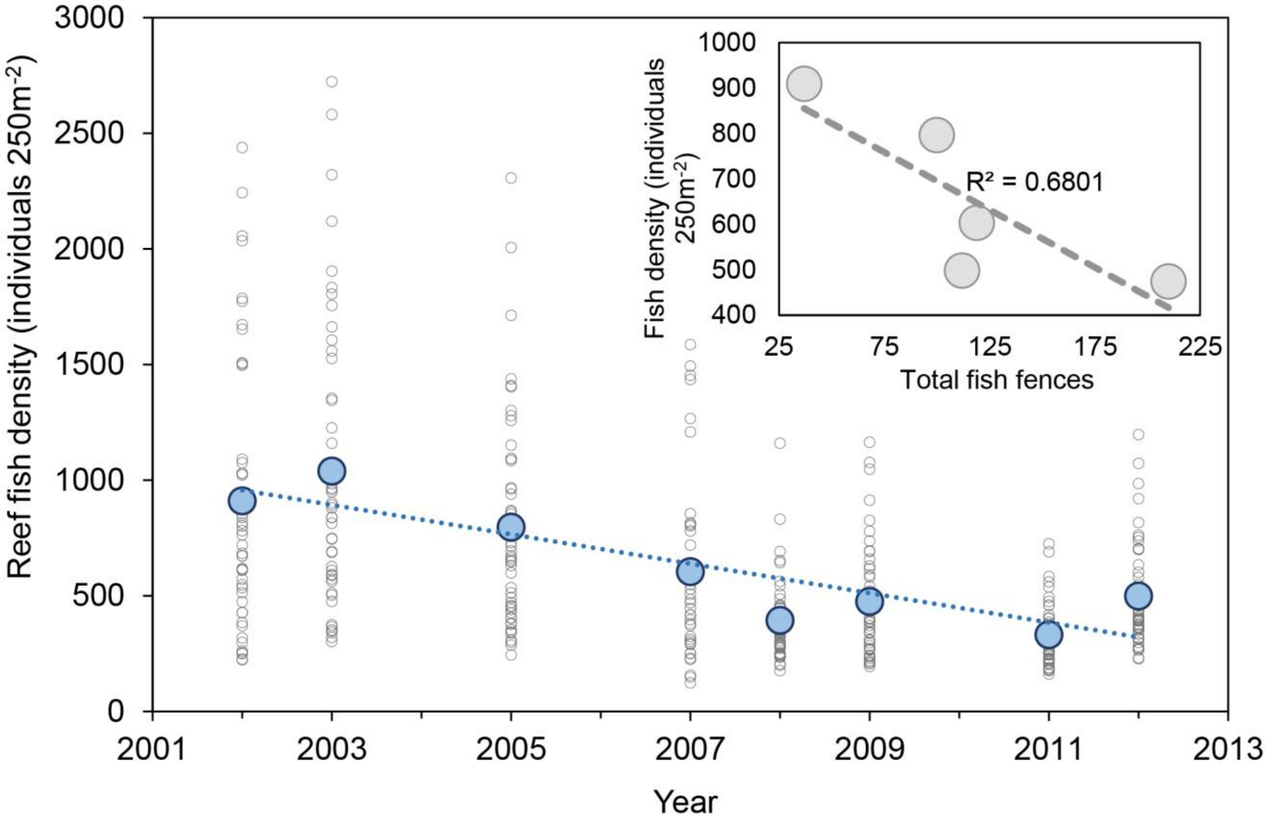

Supplementary Figure 1: Densities of reef fish in the study area between 2002 and 2012. Data were collected using underwater visual census (UVC) along 50 x 5 x 5 m belt transects via SCUBA, including all species. Six monitoring sites were visited at each time point (see Figure 1), and transects were completed in triplicate for three reef zones: flat (0-3m), crest (3-8m) and slope (8-15m). Note that one site was an offshore bank with no reef flat. Shown are individual transect values (small grey circles) and overall means for all transects, depths and sites combined (large blue), where *n* ranges from 39 to 50 transects per year. Insert shows the relationship between mean fish density and total number of fish fences in use.
