# Supplementary material for: Artisanal fish fences pose broad and unexpected threats to the tropical coastal seascape
Source: Nat Commun. 2019 May 21;10:2100. doi: 10.1038/s41467-019-10051-0 (PMC6529422; doi:10.1038/s41467-019-10051-0)
Supplement: Supplementary file 3 — Reporting Summary [file 41467_2019_10051_MOESM3_ESM.pdf]

nature research

Corresponding author(s): *Double-blind peer review submissions: write DBPR and your manuscript number here instead of author names.*

Last updated by author(s): *YYYY-MM-DD*

Reporting Summary

Nature Research wishes to improve the reproducibility of the work that we publish. This form provides structure for consistency and transparency in reporting. For further information on Nature Research policies, see Authors & Referees and the Editorial Policy Checklist.

Statistics

For all statistical analyses, confirm that the following items are present in the figure legend, table legend, main text, or Methods section.

n/a	Confirmed	
☐	☒	The exact sample size (n) for each experimental group/condition, given as a discrete number and unit of measurement
☐	☒	A statement on whether measurements were taken from distinct samples or whether the same sample was measured repeatedly
☐	☒	The statistical test(s) used AND whether they are one- or two-sided<br>*Only common tests should be described solely by name; describe more complex techniques in the Methods section.*
☒	☐	A description of all covariates tested
☐	☒	A description of any assumptions or corrections, such as tests of normality and adjustment for multiple comparisons
☐	☒	A full description of the statistical parameters including central tendency (e.g. means) or other basic estimates (e.g. regression coefficient) AND variation (e.g. standard deviation) or associated estimates of uncertainty (e.g. confidence intervals)
☐	☒	For null hypothesis testing, the test statistic (e.g. F, t, r) with confidence intervals, effect sizes, degrees of freedom and P value noted<br>*Give P values as exact values whenever suitable.*
☒	☐	For Bayesian analysis, information on the choice of priors and Markov chain Monte Carlo settings
☒	☐	For hierarchical and complex designs, identification of the appropriate level for tests and full reporting of outcomes
☒	☐	Estimates of effect sizes (e.g. Cohen's d, Pearson's r), indicating how they were calculated

Our web collection on statistics for biologists contains articles on many of the points above.

Software and code

Policy information about availability of computer code

Data collection	N/A
Data analysis	N/A

For manuscripts utilizing custom algorithms or software that are central to the research but not yet described in published literature, software must be made available to editors/reviewers. We strongly encourage code deposition in a community repository (e.g. GitHub). See the Nature Research guidelines for submitting code & software for further information.

Data

Policy information about availability of data

All manuscripts must include a data availability statement. This statement should provide the following information, where applicable:

- Accession codes, unique identifiers, or web links for publicly available datasets
- A list of figures that have associated raw data
- A description of any restrictions on data availability

The datasets presented in this study are available from the corresponding author on reasonable request.

Field-specific reporting

Please select the one below that is the best fit for your research. If you are not sure, read the appropriate sections before making your selection.

☐ Life sciences ☐ Behavioural & social sciences ☒ Ecological, evolutionary & environmental sciences

For a reference copy of the document with all sections, see nature.com/documents/nr-reporting-summary-flat.pdf

Ecological, evolutionary & environmental sciences study design

All studies must disclose on these points even when the disclosure is negative.

Study description	The study brings together multiple quantitative data sets from the case study location, primarily: (i) how fish fence effort has changed over time involving regular visual censi of total fence numbers and measurement of the dimensions and mesh size of a randomly selected subset of fences in use; (ii) changes in long term catches via monitoring of a subset of fences, specifically total weight of catch (CPUE), individuals caught, species caught and length of individuals (allowing juvenile identification); (iii) changes in long tern fish density on nearby coral reefs from underwater visual census using SCUBA; and (iv) household surveys of fishers within all local villages to determine primary gear type in use.
Research sample	Catch monitoring and fence dimension data were collected from a subset of fish fences in use at the study location each year. Selection of this subset was partially random, whilst ensuring fence owners were able and willing to return catches to a central location accessible by researchers without compromising fishing success. Local partner NGO FORKANI were responsible for liaising with the fishing community prior to researchers arriving each year and for identifying a suitable subset of fences for inclusion. Catches for each fence were then monitored regularly for a minimum of 4 weeks, ensuring a full lunar cycle was included (important considering the role of fish fences in targeting natural movements of fish).
Sampling strategy	Sample size was not pre-determined statistically. Instead, the above approach was used to identify the subset of fences to be included, ensuring the number was within the capabilities of researchers to appropriately monitor each day.
Data collection	Firstly, island-wide visual censuses of fences were conducted by circumnavigating Kaledupa and its outlying islands by boat. Secondly, the total length of each fence monitored in this study was measured along the central spine (Panaju) to provide an approximate mean total fence length for Kaledupa. Then, for catch monitoring, the total catch was weighed to provide catch per unit effort (CPUE). All fish were subsequently identified to species level, and length measurements taken of all individuals. Where more than 20 individuals of a species appeared in a single catch, a random sub-sample of 20 individuals were chosen for length measurements and mean values applied to the total number caught. Length data were used to quantify the proportion of each species and total catch caught as juveniles using published species-specific size of maturation values. For fish surveys on adjacent coral reefs, underwater visual census (UVC) was performed along 50 x 5 x 5m belt transects, with all fish identified to species level. At each site and year, triplicate transects were completed on three reef zones: reef flat (0-3m), reef crest (3-8m) and reef slope (8-15m). For household surveys, stratified randomised and semi-quantitative household interviews were conducted around the Island (spread across 17 villages) in both 2005 and 2012. For each component of this study, data were collected by a range of scientists supported by undergraduate research assistants and local partner staff.
Timing and spatial scale	Data were collected once per year (June-August) between 2002 and 2016, although not all data were collected in all years, as this study is a combination of multiple components that happened independently of one another, and each had their own unique logistical and funding constraints.
Data exclusions	No data were excluded from this study. Changes in fish length from catches over time were restricted to the ten most abundantly caught species due to the extremely high number of species observed in catches (500+), which meant that including only a subset of the most commonly caught avoided confusing the primary focus of the manuscript, whilst still acknowledging the importance of changing fish lengths.
Reproducibility	No attempts to verify the reproducibility of data were made.
Randomization	No grouping occurred as part of this study.
Blinding	Blinding was not possible in this study, as the core focus was on temporal trends, and it was impossible to blind researchers towards the year in which they were collecting data. However, as comparisons were not being made between treatments within years, we feel this should not impact the quality of data collected, not our inferences of the findings.

Did the study involve field work? ☒ Yes ☐ No

Field work, collection and transport

Field conditions	Data collection took place in situ around Kaledupa Island within the Wakatobi National Park, Indoneisa, with the support of the local communities. Conditions were appropriate for safe and efficient fieldwork, as timings were arranged to coincide with calm weather periods.
Location	Data collection took place on and around the island of Kaledupa (5°29’56.08”S 123°44’52.97”E) in the Wakatobi National Park, Indonesia.
Access and import/export	Local fishing communities were accessed with the support and guidance of local NGO FORKANI. Adjacent coral reefs and fish fences themselves were accessed using an existing boat and SCUBA logistical framework of locally operating international NGO Operation Wallacea. No samples were either imported/exported as part of this study. Data were collected as part of locally ongoing coral reef and fishery monitoring programmes supported annually by research permits obtained by Indonesian authority RISTEK.
Disturbance	No disturbance was caused by this study.

Reporting for specific materials, systems and methods

We require information from authors about some types of materials, experimental systems and methods used in many studies. Here, indicate whether each material, system or method listed is relevant to your study. If you are not sure if a list item applies to your research, read the appropriate section before selecting a response.

Materials & experimental systems

n/a	Involved in the study
☒	☐ Antibodies
☒	☐ Eukaryotic cell lines
☒	☐ Palaeontology
☐	☒ Animals and other organisms
☒	☐ Human research participants
☒	☐ Clinical data

Methods

n/a	Involved in the study
☒	☐ ChIP-seq
☒	☐ Flow cytometry
☒	☐ MRI-based neuroimaging

Animals and other organisms

Policy information about studies involving animals; ARRIVE guidelines recommended for reporting animal research

Laboratory animals	N/A
Wild animals	Fish communities were passively observed underwater for data collection on fish abundance (Supplementary Figure 1). Fishery catches were also observed to collect data on long term catch monitoring. these observations were taken from an existing active fishery, and so no catches were made specifically for the purposes of this study. Similarly, no researchers were involved in capturing or killing these animals, they were merely permitted to collect observational data on catches by the fishers involved.
Field-collected samples	N/A
Ethics oversight	No ethical approval was sought or required for the observation of in situ fish communities or fishery catches, as all data were collected passively, and no animals were captured or harmed for the purposes of this study. However, ethical approval was obtained (from Swansea University; SU-Ethics-Staff-250319/134) for the social element of this project, which involved interviewing human participants within the local fishing community.

Note that full information on the approval of the study protocol must also be provided in the manuscript.
